# Fine scale human genetic structure in three regions of Cameroon reveals episodic diversifying selection

**DOI:** 10.1038/s41598-020-79124-1

**Published:** 2021-01-13

**Authors:** Kevin K. Esoh, Tobias O. Apinjoh, Steven G. Nyanjom, Ambroise Wonkam, Emile R. Chimusa, Lucas Amenga-Etego, Alfred Amambua-Ngwa, Eric A. Achidi

**Affiliations:** 1 Department of Biochemistry, Jomo Kenyatta University of Agriculture and Technology, P.O. Box 62000, Nairobi, City Square, Kenya; 2 Department of Biochemistry and Molecular Biology, University of Buea, P.O. Box 63, Buea, South West Region, Cameroon; 3 Division of Human Genetics, Department of Pathology, Institute of Infectious Disease and Molecular Medicine, University of Cape Town, Health Sciences Campus, Anzio Rd, Observatory, 7925 South Africa; 4 West African Centre for Cell Biology of Infectious Pathogens, University of Ghana, Legon, Accra, Ghana; 5 Medical Research Council Unit the Gambia at LSHTM, Banjul, The Gambia

**Keywords:** Computational biology and bioinformatics, Genetics, Diseases

## Abstract

Inferences from genetic association studies rely largely on the definition and description of the underlying populations that highlight their genetic similarities and differences. The clustering of human populations into subgroups (population structure) can significantly confound disease associations. This study investigated the fine-scale genetic structure within Cameroon that may underlie disparities observed with Cameroonian ethnicities in malaria genome-wide association studies in sub-Saharan Africa. Genotype data of 1073 individuals from three regions and three ethnic groups in Cameroon were analyzed using measures of genetic proximity to ascertain fine-scale genetic structure. Model-based clustering revealed distinct ancestral proportions among the Bantu, Semi-Bantu and Foulbe ethnic groups, while haplotype-based coancestry estimation revealed possible longstanding and ongoing sympatric differentiation among individuals of the Foulbe ethnic group, and their Bantu and Semi-Bantu counterparts. A genome scan found strong selection signatures in the HLA gene region, confirming longstanding knowledge of natural selection on this genomic region in African populations following immense disease pressure. Signatures of selection were also observed in the *HBB* gene cluster, a genomic region known to be under strong balancing selection in sub-Saharan Africa due to its co-evolution with malaria. This study further supports the role of evolution in shaping genomes of Cameroonian populations and reveals fine-scale hierarchical structure among and within Cameroonian ethnicities that may impact genetic association studies in the country.

## Introduction

Advances in the genome-wide analysis of human genomic variation in disease states have led to the discovery of thousands of disease-associated loci^1^. Although genome-wide association studies (GWASs) have been successful in uncovering the contribution of single nucleotide polymorphisms (SNPs) to common diseases in populations with European and Asian ancestries, studies on African populations have been less successful. Population structure/substructure, characterized by extensive genetic variation and low linkage disequilibrium (LD) among and within African populations is a major cause of the poor performance of GWAS and other statistical genetics tools in Africa^2,3^.

Population structure analysis is crucial to the design, analysis, and interpretation of genetic association studies. While human genetic structure is well understood at the continental scale^4–7^, the fine-scale genetic structure of specific African populations at fine geographic ranges remains largely understudied. Previous genetic association analyses in Africa have relied on principal component analysis (PCA)^8^, with the top principal components (PCs), included as covariates in the association analyses to control for confounding by population structure^9–15^. More recently, approaches like mixed models (MM)^16–18^ and Bayesian statistics^19^ have proven particularly effective in accounting for genetic structure among and within populations in GWASs. However, given the extensive genetic heterogeneity in Africa that usually accompanies ethno-linguistic, cultural, and religious disparities, scars of genetic substructure remain ubiquitous in GWAS on the continent leading to the high false discovery rates (FDRs) and deficiency of significant SNPs from such studies^10,15^.

The human genetic diversity in Africa remains complex and multi-factorial, accrued from ancient and recent migration events. The outcome of this has been significant genetic admixture despite cultural, ethno-linguistic, and eco-geographic barriers to gene flow^7,20^. The genomes of African populations have also been shaped by evolutionary and selection pressures from infectious diseases and the environment. The relatively high frequency of the Sickle cell trait and the glucose-6-phosphate dehydrogenase (*G6PD*) deficiency in sub-Saharan Africa (sSA) perpetrated by malaria^21,22^ are classic examples of such disease pressure. Perhaps, it is the clustering of African populations into thousands of demes of local ethnicities^20^ that mirrors the extent of the genetic diversity within the continent.

In Cameroon for instance, there are over 250 tribes distributed within 3 broad ethnic groups; Bantu (BA), Semi-Bantu (SB), and Sudanese (which includes the Foulbe). A recent population genetic study revealed that chunks of the genome of individuals of the BA and SB ethnicities are shared with their African counterparts^20^. While this is expected, the ancestral relationship and extent of genetic differentiation between individuals of different ethnicities in Cameroon have not been explored. Considered the World’s most culturally diverse nation, with a complex colonial history^23,24^, Cameroon’s populations may have been subject to both genetic admixture and numerous barriers to gene flow, leading to differences in allele frequency and haplotype structure between ethnic populations. A recent severe malaria GWAS in Africa highlighted some heterogeneity in the effect and genotype frequency of some key malaria-associated loci in Cameroonian individuals relative to their sSA counterparts^15^. Hence, detailed analysis of the fine-scale structure of the ethnic groups may shed some light on specificities of the sub-populations.

This study aimed at characterizing the genetic structure of the Bantu, Semi-Bantu, and Foulbe (FO) ethnic groups from Cameroon. SNP data from 1073 individuals living in the South West, Littoral, and Centre regions of Cameroon were analyzed. The Wright’s F-statistic (F_*ST*_), model-based clustering, and PCA were used to determine the relationship between structure and ethnicity while information derived from patterns of haplotype sharing among individuals (coancestry) was used to depict fine-scale structure and ancestral admixture. Additionally, the genetic architecture of individuals in the study population was compared to those from the 1000 Genomes Phase 3 populations and genome scan was performed to map genomic regions with significantly differentiated alleles and extended haplotype homozygosity that may be due to selective forces.

## Results

### Genetic distance (F_*ST*_) and PCA show correlation with geography

**Figure 1:**
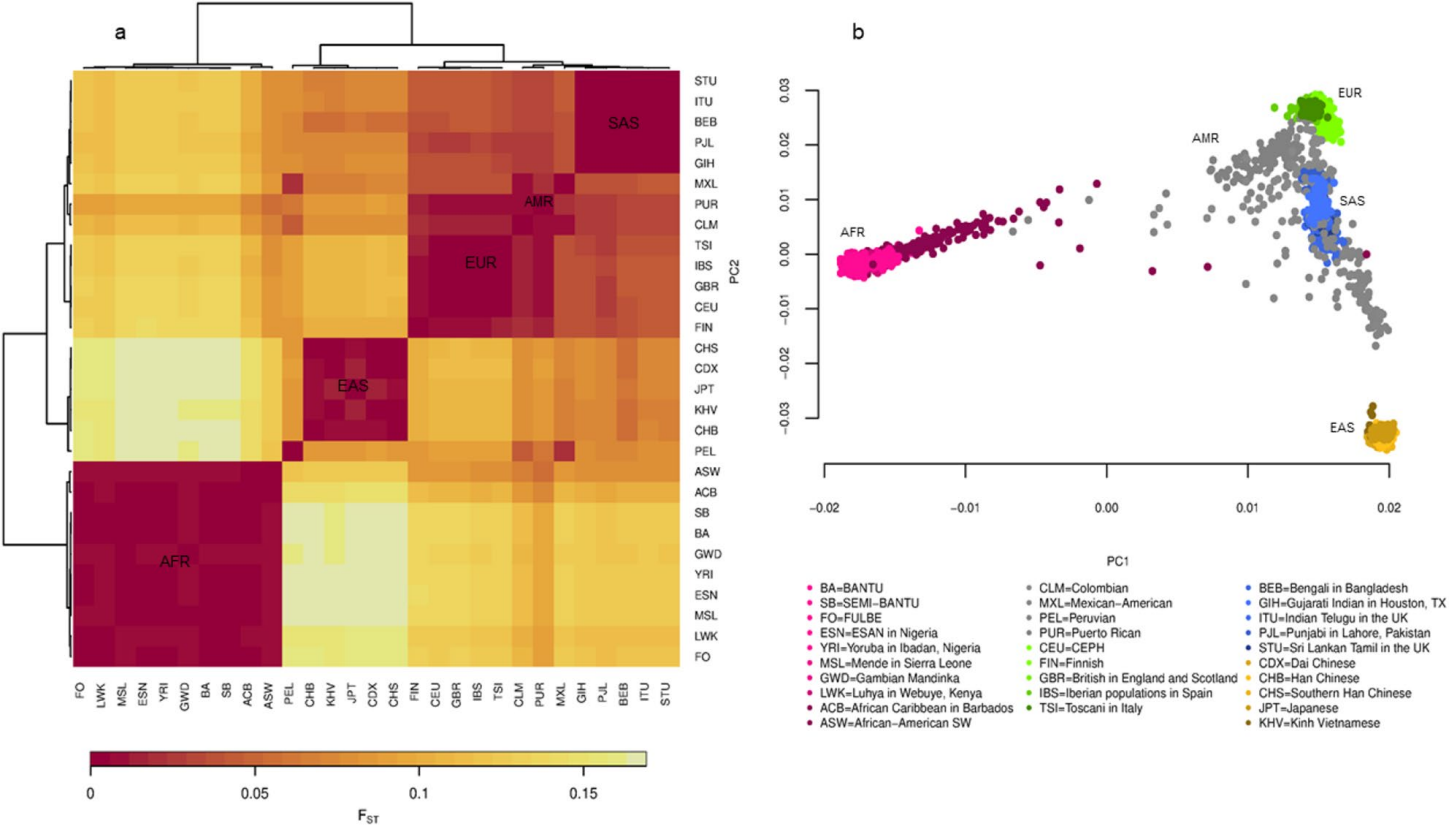
Pairwise Fst and PCA analysis of Cameroonian and world populations. (**a**) Clustered heatmap showing genetic distance by pairwise population F_*ST*_ (Hudson) estimation. AFR = African, EAS = East Asian, EUR = European, AMR = American and SAS = South Asian ancestry. The red color denotes closely related population, hence low F_*ST*_ while the decrease in redness to yellow represents increasing genetic distance (high F_*ST*_). Five clusters are apparently corresponding to the five continental proxy ancestry (distinguished broadly by five colors) in the 1000 Genomes project. (**b**) PCA of Cameroonian populations with the 1000 Genomes populations. Cameroonian populations clustered within African populations. All plots were produced using *R* 3.6.1^29^.

A total of 1073 individuals from Cameroon (BA = 492, FO = 25, SB = 556) and 2504 individuals from the 1,000 Genomes reference panel phase 3 (1KGP3) populations were studied^25,26^. F_*ST*_, PCA and other measures of genetic proximity were computed from 763,806 autosomal SNPs ascertained to be polymorphic in the Mende population from Sierra Leon (MSL) (see “Discussions” and “Methods” section) and shared (polymorphic) across all the populations. When Cameroonian populations were analyzed separately, 57,374 of these SNPs, polymorphic across all the ethnic groups, were used. We note that several estimators of F_*ST*_ have been developed and a majority of them are not adequately robust against varying population sizes. The Hudson estimator implemented in *smartpca*^27^ is, to the best of our knowledge, the only documented estimator that is immune to sample size differences and was (*smartpca version 16000*) thus employed in this analysis. Estimates for within- and among-continent population comparisons similar to those previously reported were observed (supplementary Data S1). Here, Cameroonian populations generally clustered with other African populations (Fig. 1a). The SB appeared genetically closer to the Yoruba of Nigeria (YRI) (F_*ST*_ SBvsYRI = 0.002) than did the BA (F_*ST*_ BAvsYRI = 0.003) contrary to previous estimates^20^. We further found that the FO ethnicity is relatively less genetically related to the YRI (F_*ST*_ = 0.004) compared to Cameroonian SB and BA populations. In addition, contrary to prior estimates that the Cameroonian BA population was genetically closer to the Luhya population of Kenya (LWK), we observed no difference in F_*ST*_ among the LWK population and all Cameroonian populations herein studied (all pairwise F_*ST*_ = 0.005)^20^. Interestingly, the FO, like the LWK population appeared to be more genetically close to populations of European and Asian ancestries as compared to the BA and SB, supporting possible influence of Eurasian migration back into Africa^28^. PCA generally showed positive concordance with F_*ST*_ results (Fig. 1b).

**Figure 2:**
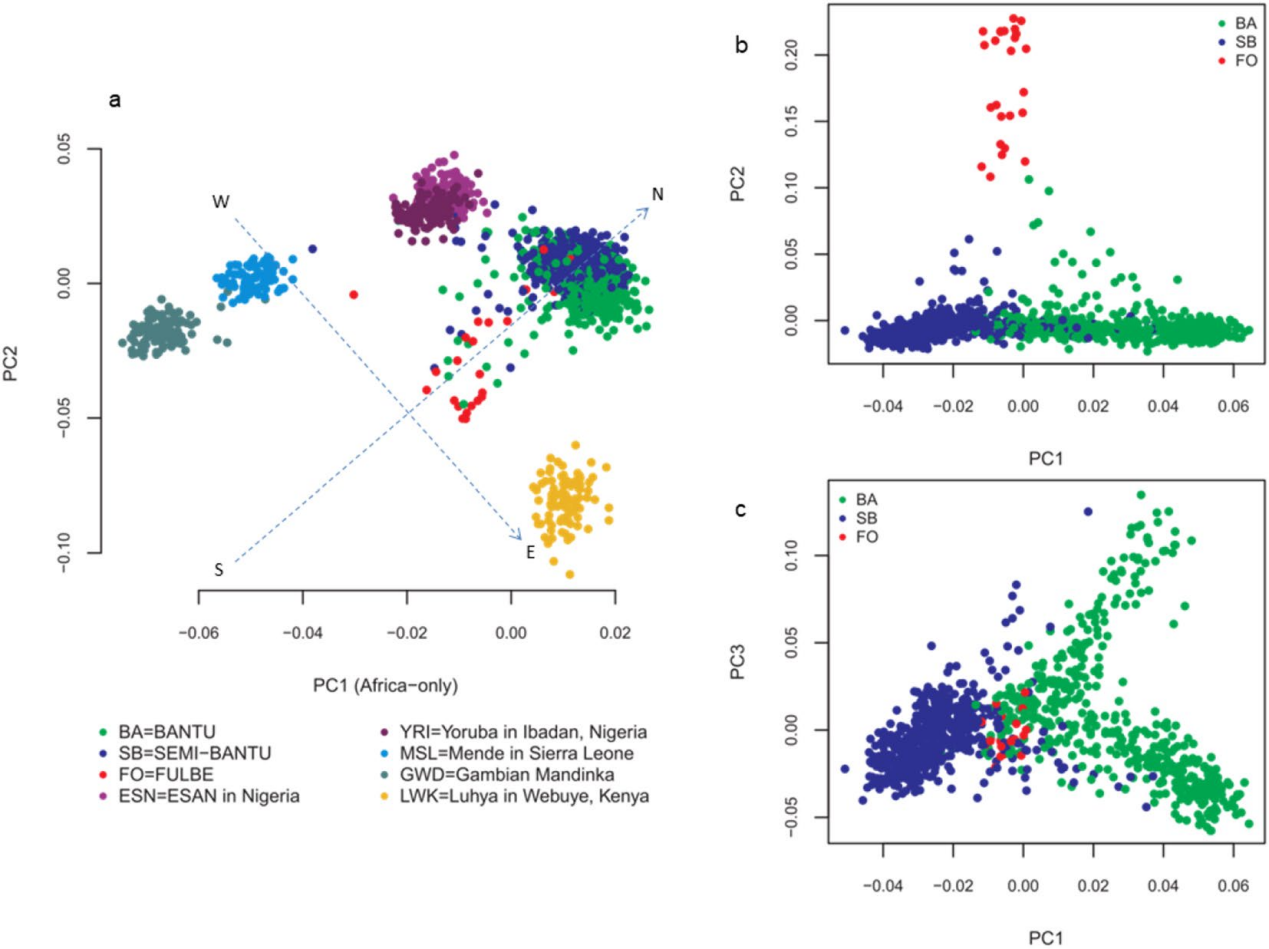
PCA of Cameroonian populations and other Africa populations. (**a**) Cameroonian populations with other African populations produce a clustering pattern correlated with geography where West African populations (GWD, MSL, ESN, YRI) clustered to the West, the lone east African population (LWK) clustered to the East, while Cameroonian populations clustered West-Centrally. (**b**) PCA for Cameroonian ethnicities only. PC1 and PC2 separate the three ethnicities, (**c**) PC1 and PC3 separate the Bantu and Semi-Bantu. All plots were produced using *R* 3.6.1^29^.

In an Africa-only analysis, Cameroonian populations clustered between Nigerian and Kenyan populations based on principal components 1 (PC1) and 2 (PC2) (Fig. 2a). There was a general West–East cline (adjusted axes) consistent with the concept of isolation by distance (geographic location). Cameroonian populations clustered West-Centrally reflecting their location on the continent. Initial PCA with *smartpca*’s default 5 outlier removal iterations (*numoutlieriter*) resulted in ancestry outliers (Supplementary Fig. S1) which were eventually removed by increasing the *numoutlieriter* to 10. Cameroonian ethnicities could already be distinguished at this point, although a high degree of spread remained apparent. In addition, FO individuals differentiated from the BA and SB, with SB clustering closer to Nigerian populations than the other ethnicities.

Further PCA of only Cameroonian ethnicities revealed three clusters (Supplementary Fig. S2). To increase the resolution of the clusters, we ran PCA with SNPs from *smartpca*’s pairwise F_*ST*_ ≥ 0.003 classified here as “ancestry informative markers” (AIMs). In principle, AIMs are loci with substantial allele frequency difference across populations^30^. They can be used to assign individuals into clusters based on biological (ancestral) or geographical (continental) boundaries^31^. Although pre-calculated lists of AIMs exist, they mostly apply to cross-continental populations^32^. Furthermore, there are several measures of marker information content as listed in Table 1 of Rosenberg et al.^31^. We therefore used the F_*ST*_ measure in this study to generate AIMs by selecting all SNPs with F_*ST*_ greater than or equal to the highest F_*ST*_ (genetic distance) between Cameroonian ethnic groups. F_*ST*_ between the BA and SB was 0.001 whereas F_*ST*_ between the FO and both BA and SB was 0.003. Hence, AIMs were considered as SNPs with pairwise population F_*ST*_ (Hudson) ≥ 0.003. This strategy saw an increased resolution of the clusters, clearly separating the FO from the BA and SB based on PC2 (Fig. 2b). Furthermore, a substructure within the FO population was resolved, showing two separate clusters. At fine scale, using AIMs, a substructure within the BA population based on PC1 against PC3 was resolved (Fig. 2c). We note that increasing the F_*ST*_ at which AIMs were selected only further separated the FO from the BA and SB without achieving further resolution of the BA and SB clusters.

**Table 1:** Variants with strong signatures of selection in coding genomic regions sorted by ihs scores from most negative (top) through least negative to most positive (bottom).

rsid	chr:pos	ref	alt	alt.AF	ihs	*p*-value (bh)	*a.a change*	gene
rs10947368	6:32975341	C	T	0.1076	− 6.422	5.73e−05	K120N	*HLA-DOA*
rs8192564	6:32191822	G	A	0.06011	− 5.494	0.0024	–	*NOTCH4*
rs115261305	6:32793668	C	A	0.1319	− 5.385	0.0030	–	*TAP2*
rs1126544	6:33037061	G	C	0.1281	− 5.133	0.0085	T121T	*HLA-DPA1*
rs3800326	6:28264717	C	T	0.1039	− 5.111	0.0089	P256L	*PGBD1*
rs61737338	6:28227217	C	T	0.08388	− 4.922	0.0154	S23F	*NKAPL*
rs7943508	11:57003581	C	T	0.0657	− 4.861	0.0176	V300I	*APLNR*
rs6582601	12:38716034	C	T	0.1253	− 4.850	0.0183	–	*ALG10B*
rs2233954	6:31105672	G	A	0.08854	− 4.802	0.0207	–	*PSORS1C2*
rs34304311	6:28093263	G	A	0.07549	− 4.657	0.0298	L14L	*ZSCAN16*
rs6115256	20:25666642	C	T	0.1771	− 4.600	0.0348	L48L	*ZNF337*
rs17190762	6:31126992	G	A	0.06291	− 4.528	0.0429	–	*TCF19*
rs10896290	11:56128081	A	G	0.2679	− 4.524	0.0429	Y120C	*OR8J1*
rs61729683	6:32185818	C	T	0.06337	− 4.522	0.0430	A526A	*NOTCH4*
rs78133850	11:57004659	G	A	0.06897	− 4.508	0.0442	–	*APLNR*
rs73468666	11:56958933	G	A	0.1761	− 4.493	0.0462	–	*LRRC55*
rs75301276	11:55944198	C	T	0.06058	− 4.491	0.0462	Y35Y	*OR5J2*
rs58567530	16:48172185	C	A	0.06943	− 4.489	0.0464	L311L	*ABCC12*
rs3013106	1:13802437	G	A	0.4455	4.995	0.0116	S254S	*LRRC38*

rsid, Reference SNP ID; chr:pos, Chromosome number and position; ref, Reference allele; alt, Alternate allele; alt.AF, Alternate allele frequency; ihs, Integrated haplotype score; *p*-value (bh), Benjamin–Hochberg adjusted *p*-value; a.a change, Amino acid change.

### Distinct ancestral proportions and fine structure among Cameroonian ethnicities

**Figure 3:**
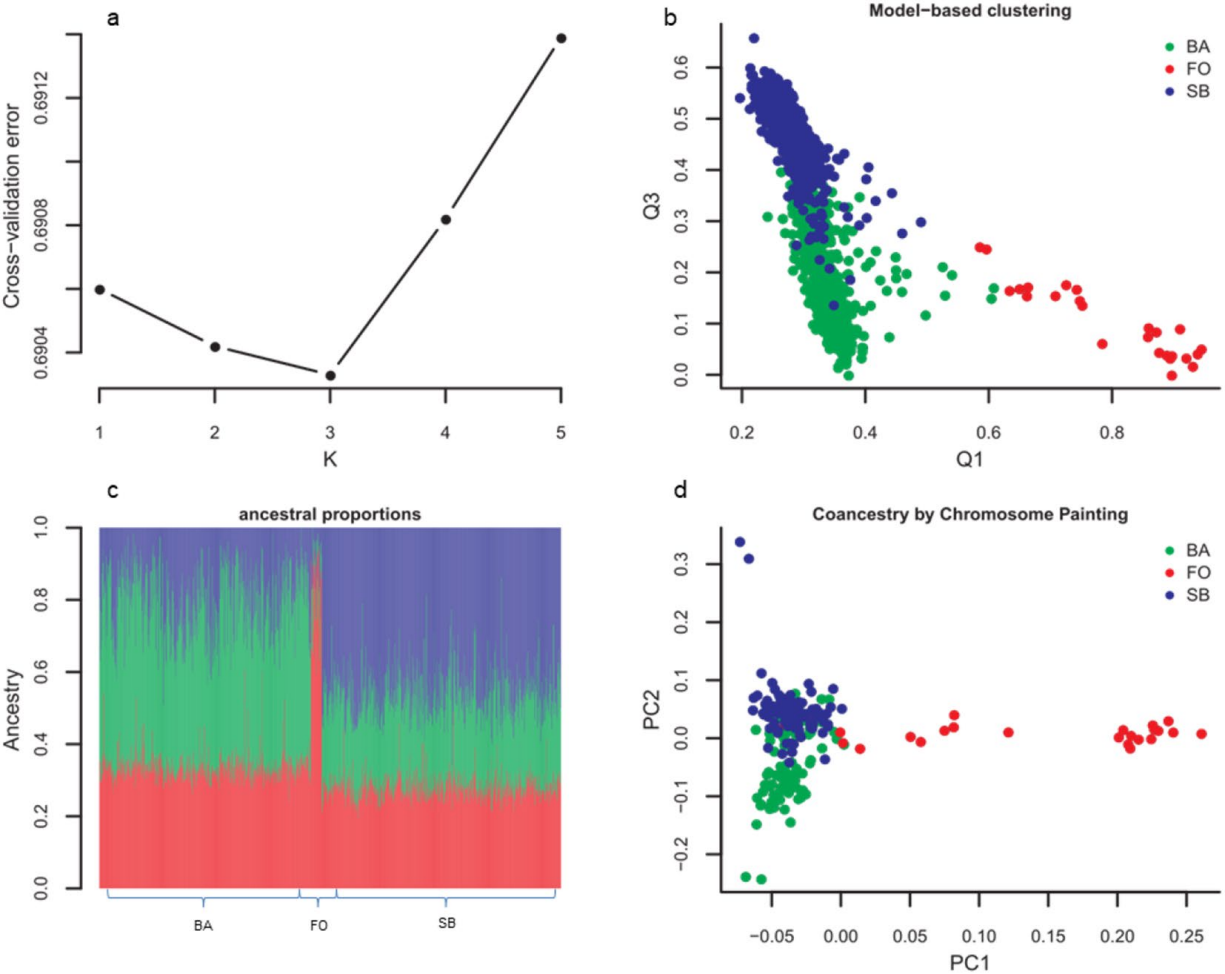
Model-based clustering and Coancestry estimation. (**a**) Model-based clustering cross-validation (CV) error. Lowest CV error recorded at k = 3 indicating three clusters. (**b**) and (**c**) show ancestral proportions Q, for each ethnicity colored using the RGB color scheme. (**d**) Coancestry estimation by FineStructure. All plots were produced using *R* 3.6.1^29^.

Using a total of 81,415 high quality independent AIMs (MAF > 0.05, LD < 0.2), we computed ancestral proportions (Q estimates) for our three ethnic groups with five k-parameters using model-based clustering implemented in Admixture^33^. At K = 2, model-based clustering differentiated the three ethnicities albeit with low resolution. However, at K = 3 where the lowest cross-validation error was recorded (Fig. 3a), the three ethnicities were clearly differentiated (Fig. 3b). Ancestral proportions (Q) estimated [green predominant in the BA (~ 45%), red in the FO (~ 75%) and blue in the SB (~ 45%)] (Fig. 3c) show that the different ethnic groups differ by allele frequencies (Supplementary material) and/or haplotype structure (see below). When we applied *Structure v2.3.4*^34^ in the admixture mode using 3 pre-defined clusters on 50 BA, 50 SB, and 25 FO individuals setting 20,000 burnin and 100,000 main iterations, the ancestral proportions were resolved even better (FO = 76.6%, BA = 73.3%, and SB = 68.4%) (Supplementary Fig. S4). Here, the FO appeared to harbor a large chunk of ancestry from a non-Cameroonian source.

While single marker approaches that measure genetic distance usually require markers in approximate linkage equilibrium (unlinked), haplotype-based approaches have the potential to exploit the linkage information in genotype datasets to estimate genetic distances with finer resolutions. Therefore, we used a haplotype-based approach of coancestry estimation by “chromosome painting” implemented in fineStructure^35^ for fine scale resolution of Cameroonian ethnicities. In this analysis, we thinned the samples using Plink1.9′s*—thin-indiv-count* (which removes samples at random until only the required number is left) to obtain 50 BA and 50 SB individuals together with the initial 25 FO individuals to make a final analysis set of 125 individuals. We observed isolation of a subgroup of FO individuals from the BA and SB by PC1 (Fig. 3d). PC2 separated the Bantu and Semi-Bantu, with significant numbers of BA individuals clustering with SB. Generally, all the ethnicities showed a cline into a central cluster that appeared to be a set of admixed individuals, consistent with their longstanding cohabitation while their separation suggests some evidence of ancient genetic isolation and/or gene flow from other populations. The three extreme clusters may represent individuals with the basal ancestry for each respective ethnicity.

### Selective sweeps target genes associated with response to disease

**Figure 4:**
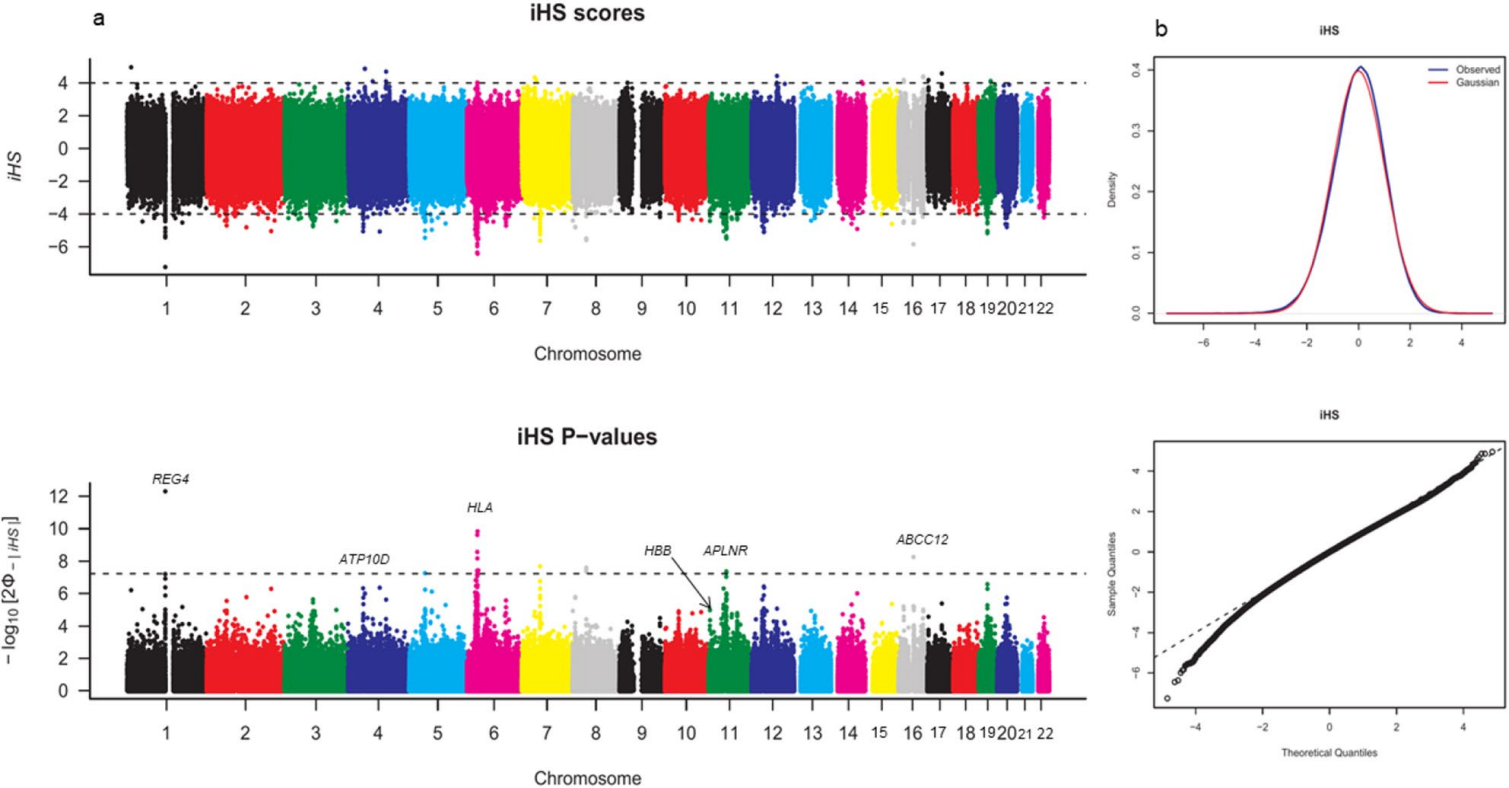
iHS and corresponding –log10(*p*-values) Manhattan plots. (**a**) iHS plot for the autosomes. Negative values signify selection on derived alleles while positive values are associated with selection on ancestral alleles. (**b**) Distribution of iHS values as observed in the populations (blue) and as expected under neutral evolution (red). Lower plot represents quantile–quantile (Q–Q) plot of iHS *p*-values. The plot shows that the test statistics are not inflated. The plots were generated using the R package *qqman*^45^.

Genomic regions under positive selective sweeps are usually characterized by extended haplotype homozygosity (EHH) and low genetic diversity. A genome-wide scan for such regions harboring signatures of selection by the standardized integrated haplotype score (iHS) which measures the EHH identified strong signatures on multiple chromosomes. This included missense and regulatory region variants in genes overwhelmingly associated with response to infections. The scan identified a total of 133 SNPs within 57 overlapped genes and 173 overlapped transcripts across chromosomes 1 to 12, 14, 16, 17, 19, and 20 with significant signatures of selection at iHS threshold of ± 4 (Fig. 4a). Chromosome 6 and 11 harbored the highest proportion of variants involved in selection. Table 1 shows the variants with strong signatures of selection that occurred in coding regions. The entire list of 133 selected variants can be found as Supplementary Data S2 online along with the corresponding variant effect predictor (VEP)^36^ and BioMart-annotated gene list. Although the strongest signature occurred on chromosome 1 around the *REG4* gene (iHS = − 7.23, *p*-value = 4.67 × 10^−13^), the most consistent signatures were recorded on chromosome 6 spanning the HLA region which has been reported in several previous studies of selection^37–40^. The SNP rs10947368, a missense variant on *HLA-DOA* emerged with the strongest signal within the HLA region (iHS =  − 6.42, *p*-value = 1.38 × 10^−10^) (Table 1). In addition, suggestive signatures of selection were recorded in the hemoglobin-beta (*HBB*) gene cluster of chromosome 11, a region with longstanding knowledge of balancing selection under the influence of malaria^41,42^. However, the strongest signal on chromosome 11 was a relatively uncommon missense variant (rs7943508) in the *APLNR* gene, implicated in hypertension and some cancers^43,44^. Generally, selection signatures were not isolated, but occurred in clusters of consecutive SNPs positions, consistent with “genetic hitchhiking” around a positively selected variant, typical of selective sweeps. Our genome-wide significance threshold was estimated at 6.033 × 10^−08^ (see “Methods” section). The iHS values obtained were generally normally distributed as expected under neutral evolution with a slight deviation from the expected distribution reflecting the regions showing evidence of selection (Fig. 4b).

### Allele frequency differences due to selection pressures among Cameroonian ethnicities

Allele frequency differences among populations can be caused by several factors including random genetic drift following isolation, whether by distance, or more commonly in Africa, by cultural and religious disparities (sympatric differentiation). Also, allele frequency differences may arise due to changes in population dynamics like rapid population growths or contractions, and selection pressures among others. Therefore, to isolate only selection pressure effect on allele frequency differences among Cameroonian ethnicities, we computed the extended Lewontin–Krakauer F_*ST*_ outlier statistic (FLK) which accounts for unequal effective population sizes (Ne) and hierarchical population structure^46^. We used the MSL population as an out-group as earlier mentioned. Several genomic regions with subtle allele frequency differences between the ethnicities were observed although none of these regions remained significant after correction for multiple testing by the Benjamin–Hockerbeg method^47,48^. However, evidence of positive selection remained apparent in the HLA region on chromosome 6. Specifically, positions on chromosomes 2, 6, 8, 10, 17, 18, and 22 were subtly differentiated among the ethnicities (Supplementary Fig.S5 & Supplementary Table S1). We note that the FLK test is not robust to low levels of heterozygosity and it was shown to perform poorly (with a high false positive rate) at SNPs with ancestral allele frequency (AAF) < 0.2 and AAF > 0.8 (that is less common SNPs and SNPs nearing fixation)^46^. Therefore our analysis was based on SNPs within the AAF range of 0.2–0.8 (see “Methods” section). A cluster-stratified analysis of the derived allele frequencies revealed a great heterogeneity in the frequency spectra of the three ethnic groups at selected loci (Supplementary Fig. S6).

### Genes involved in food/drug metabolism targeted by selection

**Figure 5:**
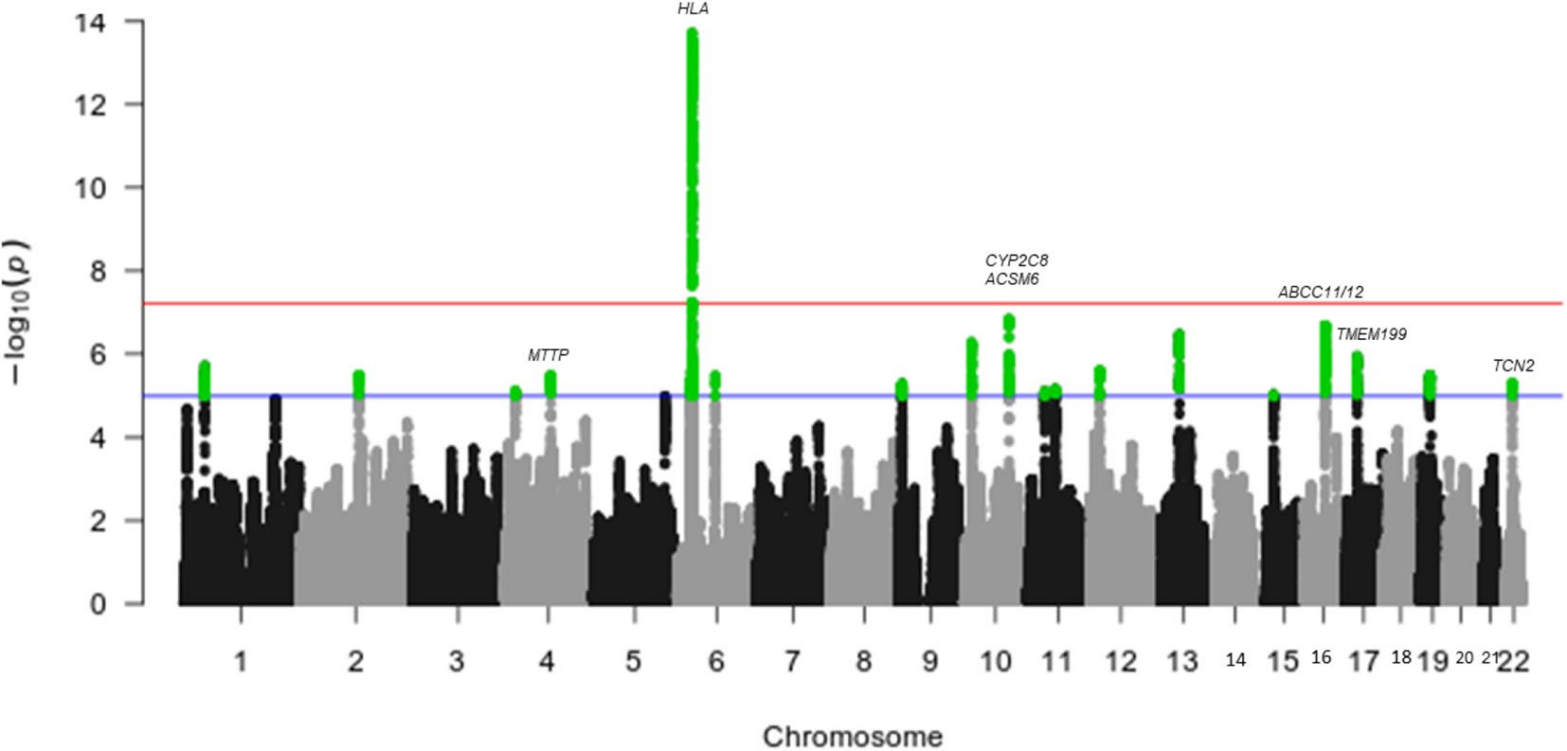
Manahattan plot of hapFLK *p*-values. Genome-wide significance threshold (red line), suggestive threshold (blue line). The plot was generated using *qqman*^45^.

Given that genomic regions under strong positive selection usually pull along nearby SNPs (genetic hitchhiking), we screened for selection using clusters of loci (haplotypes) by the haplotype variant of the FLK test (hapFLK). This resulted in a strong signature on chromosome 6 as was recorded by iHS, while several other regions on multiple chromosomes showed suggestive signals (Fig. 5). Of note were signatures on chromosomes 10, 16, 17, and 22 occurring in genes associated with food/drug metabolism. We observed signals on chromosome 10 associated with missense variants on the *ACSM6* gene associated with acetyl coenzyme-A production (*p* = 1.62 × 10^−06^), and on the *CYP2C8* gene, a cytochrome P450 superfamily enzyme member associated with drug metabolism (*p* = 6.09 × 10^−06^). Multiple missense variant signals were also observed in the *ABCC11/12* gene on chromosome 16 (*p* = 5.39 × 10^−07^), an ATP binding cassette subfamily member involved in multi-drug resistance. In addition, signals were observed on the *MTTP* gene on chromosome 4 (*p* = 3.73 × 10^−06^) involved in triglyceride transfer and lipoprotein assembly, the *TMEM199* gene on chromosome 17 (*p* = 5.35 × 10^−06^) whose deficiency is associated with abnormal glycosylation^49^, and the *TCN2* gene on chromosome 22 (*p* = 6.76 × 10^−06^) involved in the absorption of vitamin B12 (cobalamin). Our threshold for genome-wide significance was estimated at 6.02 × 10^−08^ (see “Methods” section).

### Extant Cameroonian ethnic populations may be under differential selective pressure

To further investigate which genomic positions are differentially selected among the ethnic groups, we performed a cross-population selection scan using the Rsb statistic implemented in the REHHv3.0.1 R package^50,51^. We found chromosome 6 to be strongly selected in the BA and SB and only subtly selected in the FO. This could be due to the low sample size of the FO population. The BA population showed additional signatures on chromosome 6 involving the missense variant rs9276 on the *HLA-DPB1* consistent with previous reports of selection in this gene in Bantu-speaking African populations (BSP)^52^ and the variant rs1419638 on the *OR5V1* gene, as well as on chromosome 7 unique to the BA ethnic group. Likewise, the SB showed specific signatures on chromosomes 16 and 20, while strong signatures specific to the FO population were recorded on chromosomes 1, 7, 9, 10, 16, and 19 (Supplementary Fig.S8a-d). Again, these selection signatures primarily implicated genes involved in disease response.

## Discussion

An in-depth understanding of population structure is critical in genetic studies, particularly in highly diverse and admixed African and American populations because of its potential to impact genetic association studies that usually have small effect sizes and rare variant analyses^53,54^. Characterizing the genetic architecture of specific populations is thus a “higher level of priority” under the “Basic Genomics and Genomic Technologies” focus area of the NHGRI (https://www.genome.gov/about-nhgri/strategic-plan). This study sought to ascertain the fine-scale genetic structure in Cameroon by analyzing the genotype of 1073 individuals from the South West, Littoral, and Centre regions.

The subtle differences in pairwise F_*ST*_ observed among Cameroonian ethnic populations sampled in this study indicates substantial mixing and common recent coancestries. However, the fact that the estimates were non-zero values supports the existence of distinct ancestral proportions. The FO (belonging to the Sudanese ethnic division) appear to have North African roots related to Fulani migration from north eastern Nigeria and Chad. The BA, on the other hand, are thought to have been the earliest inhabitants of Cameroon, with traces of their ancient civilization still prominent in the pigmies of the South and East. In fact, some studies have associated the spread of the BA ancestral proportions found in Central, South and East Africa to a Bantu expansion that originated somewhere around South Western Cameroon^20,55–57^. The SB mainly inhabit the Western highlands and grass fields of the West and North West of Cameroon. Together with the BA of the South West, the SB of the North West also appear to have endured a four-decade complex cohabitation with Eastern Nigerian populations during colonial era^23^. Therefore, the ancient interactions, and interactions of the recent past of Cameroonian populations with other populations may have paved the way for substantial genetic admixture and drift.

The close similarity in ancestral proportions among the BA and SB is expected (Fig. 3a). The dissimilarities may be attributable to many factors including varying degrees of contact with different external populations with subsequent genetic drift as could be have been possible during their pre-colonial and colonial era as well as following different selective pressures. According to historical records, Portuguese slave-trading on the South-Western coast of Cameroon began as far back as the fifteenth century and was followed by British commercial hegemony in palm oil and ivory in the nineteenth century, and an eventual German sovereignty in 1884 that was succeeded by French and British rule after World War I^58^. Following these events, parts of Nigeria, Chad, Central African Republic, Congo, Gabon, and Equatorial Guinea today were parts of Cameroon under the German and subsequently French and British rule^23^. These were therefore good recipes for genetic admixture and drift.

The considerable dissimilarity in the ancestral proportion of the FO ethnicity from the others is not surprising as was apparent in their allele frequency distribution. However, their splitting into two distinct clusters may provide evidence of genetic heterogeneity within the ethnic group. They are believed to have northern African roots associated with the ancient Sao civilization that flourished around the shores of Lake Chad around ninth–fifteenth century AD, and a Hausa-Fulani land invasion from Nigeria by the 1800s that led to the establishment of a large Islamic empire involving much of the northern regions of Cameroon. Given the barriers imposed by religious differences, as much of the South of Cameroon practiced Christianity^23^, it is possible that some isolation of the populations was automatically imposed. Large disparities in ancestral proportions among the FO and the BA and SB ethnicities may, thus be due to a longstanding barrier to gene flow due to a sympatric differentiation among the populations (That is they live together but do not interbreed to a great extent). Such a relationship has been documented in the Malian and other African populations^59–64^. The separation of the FO ethnicity into two distinct clusters by chromosome painting, one close to the BA and SB and the other quite apart, further indicate that the FO is not homogeneous due to some limited but detectable coancestry with the other ethnic groups. Indeed, it is known that non-FO individuals integrated in to the Fulani–Hausa populations as herdsmen and these may have contributed to the admixed FO sub-group.

We, however, note that such a retrospective stochastic assessment of the genetic differences among Cameroonian ethnicities and other populations with respect to their demographic histories has several limitations; first, our analysis relied on self-reported ethnicity of the father and mother of each participant which may not have been accurate. Second, not only the SB of the Western Highlands cohabited with Nigerian populations during the British and French sovereignty, but the BA population of the South West as well. Perhaps, other factors may have been in play to account for the close genetic relatedness between the SB and the YRI of Nigeria. Nevertheless, these results highlight key differences in the genetic architecture of Cameroonian ethnicities that may have significant bearing on genetic association studies for this population. For instance, the fine-scale clustering of the three ethnicities based on information provided by patterns of ancestral haplotype sharing^35^ indicates that different haplotype structures may underlie the various ethnicities and consequently different linkage disequilibrium and recombination patterns. Given that genotype imputation, which is constantly employed to boost the power of association studies, relies upon linkage disequilibrium between SNPs^9,19,65,66^, it may be inferred from this analysis that the different populations would perform differently on imputation^3^. Therefore, to optimize the imputation on Cameroonian datasets, an imputation panel including individuals across Cameroon’s ethnic groups, or a panel—as that recently provided by the NHLBI’s Trans-Omics for Precision Medicine (TOPMed) program^67^—including individuals with close genetic ancestry to Cameroon’s ethnic populations would be required. In addition, considering that these genetic differences would characterize different axes of genetic variation in an association study, one would expect significant false positive results when all the ethnicities are analyzed together. Therefore, larger sample sizes would be required for association studies in such a highly structured population to be sufficiently powered to identify markers associated with specific phenotypes. Hence, association analysis performed on each ethnicity separately would be more profitable given that ethnic information is accurately captured.

Genetic association studies involving Cameroonian populations have reported disparities in the association pattern of SNPs in Cameroonians when compared to other sub-Saharan African (sSA) countries. In their recent study^15^, the sickle cell trait (HbAS), encoded by the rs334 locus, known to contribute the strongest protective effect against severe malaria in sSA, was found to have its weakest effect in Cameroon. One would have expected a high protective effect at this locus in the Cameroonian populations, owing to the country’s high sickle cell disease (SCD) burden^68^. Although the complex polygenic nature of malaria and environmental factors may be at play, it is possible that the prevalence of the HbAS trait differs significantly among Cameroonian ethnicities such that a joint analysis could have masked the real effect. More so, MalariaGEN’s analysis was based on the BA and SB ethnicities of Cameroon only. Interestingly, the FO ethnicity is more genetically close to Gambian populations (supplementary Data S1) reported to have the highest protective effect against severe malaria^15^. Hence this may be a classic example of the potential for population structure to bias allele frequency distribution and affect the outcome of GWA analysis.

Population genetic approaches that measure genetic distance and quantify shared ancestry are more robust when SNPs are ascertained to be polymorphic in an out-group^7,8,27^. However, out-group ascertainment in African populations remains a challenge as the “most recent common ancestor” of African populations remains to be established. Although the roots of anatomically modern humans have recently been traced to Botswana^69^, the Mende population from Sierra Leone (MSL), shown to harbor the largest proportion of ancestry from a basal African lineage^7^ fitted well as an out-group in our analysis. A couple of test analyses supported this observation; (1) pairwise F_*ST*_ estimates with the 1000 Genomes populations without SNP ascertainment required either over a million SNPs or > 50,000 SNPs with MAF > 0.35 to observe estimates similar to those previous reported, (2) SNP ascertainment with all African populations except the MSL did not result in F_*ST*_ estimates as have been previously reported (3) Finally, SNP ascertainment in the MSL population resulted in similar F_*ST*_ estimates as have been previously reported using less than a million SNPs with MAF as low as 0.05. Moreover, the MSL population was estimated to have differentiated ~ 300 thousand years ago (ka)—200 ka^7^, about the same time modern humans are thought to have originated from Botswana (Southern Africa)^69^. Hence, in the absence of a publicly available and well-established out-group for African populations, the usage of the MSL population may serve such a purpose.

Finally, we observed signatures of selection in this study, suggesting that Cameroonian populations have come under strong disease pressure. The strong signatures targeted primarily immune response and food/drug metabolism genes, suggestive of polygenic adaptation of the population to diseases and changes in diet. Selection, therefore, acts on multiple loci across multiple genes to simultaneously drive phenotypic adaptation^7^, although one would expect, in principle, the core locus affecting a particular trait to be under a selective sweep. Indeed, African populations have had to endure immense pressure from infectious diseases being the oldest populations of anatomically modern humans (formerly hunter-gatherers)^6,7,20^. The case of malaria and SCD is well established, and both conditions are thought to have emerged around the same time (4000–5000 years ago) coinciding with the adoption of agriculture in Central Africa^70^. Recent reports however suggest that the human *Plasmodium falciparum* malaria and SCD arose much earlier, and at different times; ~ 60,000 and ~ 22,000 years ago respectively^42,71^. We also observe weak signatures of selection in the hemoglobin-beta (*HBB*) gene cluster. It is expected to pick weak signatures of selection in this region by iHS (which mostly targets positive selection) in particular since the method looks at the homozygosity of clusters of SNPs^72^. Signatures of balancing selection would appear weak because, although alleles occur at intermediate frequencies, an extended homozygosity of alleles of the same type as the core/focal allele would signify selection.

Meanwhile, as SNP-based approaches for selection scan continue to yield useful information regarding the adaptive evolution of specific genomic loci, valuable information may still be missed at loci with more complex genetic architectures. This is particularly true for regions with structural variants and weak linkage disequilibrium such as the Dantu [*GYPA-B*] rearrangement in the glycophorin gene cluster, a region of ancient balancing selection^73^ and the common alpha-thalassemia deletions^74^, both of which are prevalent in malarious areas^75–77^. Therefore, novel tools that incorporate such variants in selection scans would be highly beneficial.

## Conclusion

Population structure remains a major confounding factor in genetic association studies, diminishing the power of GWAS in Africa. Smarter sampling strategies and analysis designs are therefore needed to effectively detect and significantly minimize/correct population structure effects in African populations. We observe considerable substructure within our Cameroonian populations to warrant that GWAS is performed on an ethnicity basis. In addition, the analysis of a larger number of Fulani individuals in Cameroon with their Bantu and Semi-Bantu counterparts may shed further light on the contribution of their possibly non-Cameroonian ancestral proportions and the broader demographic history of this West-Central African region. The characterization of the genetic structure within other African populations will elucidate the real extent of their effects so as to inform genetic association studies on the continent.

## Methods

### Samples and genotyping

Our analysis was conducted with samples from unrelated individuals belonging to the Bantu, Semi-Bantu and Foulbe ethnicities from the South West, Littoral, and Centre regions of Cameroon. The individuals were recruited (1471) as part of a malaria cross-sectional study in Cameroon between 2003/05 and 2007/08 and DNA extraction was performed as previously described^78^. The samples were then contributed to the Malaria Genomic Epidemiology Network (MalariaGEN) Consortial Project 1 (CP1) where genotyping, genotype calling, and alignment to the human reference genome build 37 (hg19) were performed as previously described^10^. Data access, retrieval and analysis were performed according MalariaGEN policies^79^.

### Quality control

Sample QC was performed on the autosomes and the X chromosome (of the 1471 samples) using Plink1.9^80^. We explicitly removed individuals whose reported nationality was “Non-Cameroonian” or “Missing”. Individuals with discordant sex information were identified and excluded using Plink1.9′s*—heck-sex* filter. Furthermore, individuals with more than 10% missing data rate (missingness), and individuals with outlying heterozygosity were excluded from further analysis. Related samples were excluded by computing an identity-by-descent (IBD) matrix with 88252 high quality independent SNPs (MAF > 0.35, SNP missingness < 5%), LD-pruned such that all pairs with correlation, r^2^ > 0.2 within any 5 kb region, using a step size of 50b, were excluded along with all those that failed the Hardy–Weinberg equilibrium (HWE) test at *p*-value 1 × 10^−8^. One individual from each pair with Pi_HAT (mean IBD) > 0.1875 (halfway between expected IBD for third- and second-degree relatives) were removed, removing only the individual with a higher missingness (Fig.S1) and resulting in 1185 individuals. To further exclude individuals of outlying ancestry, we projected Cameroonian populations against African populations of the 1000 Genomes reference panel phase 3 (1KGP3)^81,82^ using *smartpca version 16000* (Cambridge, MA, USA) from the EIGENSOFTv7.2 package^8^. This saw the removal 111 individuals, resulting in a final set of 1073 individuals. SNP quality control and subsequent analyses were performed on autosomal SNPs (2,261,351). SNPs with minor allele frequency (MAF) < 1%, missingness > 4%, and SNPs that showed significant deviation from the HWE at *p*-value < 1 × 10^−8^ were excluded. A total of 1,588,393 autosomal SNPs passed all QC procedures and were retained for further analysis.

### Phasing

Prior to phasing, we excluded palindromic A/T, C/G SNPs which are usually problematic to the procedure. We then used the WRayner perl script v4.2.9 (https://www.well.ox.ac.uk/~wrayner/tools/) to check and validate the dataset against the 1KGP3 for ref/alt allele assignment, ID names, positions and alleles. VcfCooker v1.1.1 (https://github.com/statgen/gotcloud/blob/gotcloud.1.17.5/bin/vcfCooker) was used to convert the dataset from plink binary format to VCF format, while checking the validity of the dataset against the human reference genomic build 37. To further ascertain for ancestral/derived allele assignment, we used Plink2^80^ to scrap ancestral allele assignments from ENSEMBL genome’s 1000 Genomes Phase 3 release 98 variation file downloaded from ftp://ftp.ensembl.org/pub/release-98/variation/vcf/homo_sapiens/ (Accessed 2019-09-28)^83^. We sorted the dataset according to base-pair position with bcftools v1.9 from the SAMtools package^84^ and phased each chromosome separately with EAGLE v2.4.1^85^ against the 1KGP3 reference panel, using the combined HapMap recombination map in build 37 coordinates included in the software. We used a k parameter (–Kpbwt) of 50,000 (default 10,000) conditioning haplotypes and 10 iterations (–pbwtIters) to increase the phasing accuracy. Phasing accuracy was assessed from the log file produced by the software.

### F_ST_ estimation

Prior to F_*ST*_ and PCA analyses, we LD-pruned the SNPs such that pairs with r^2^ > 0.1 within a window of 5 kb were pruned out using a step size of 50 bp. In addition, we excluded SNPs with MAF < 0.05. Pairwise F_*ST*_ estimates among the three ethnic groups within our study populations and among our study populations and the 1KGP3 reference populations were computed using *smartpca*. The tool computes the Hudson estimator of Wright’s F_*ST*_ which is immune to sample size differences, and uses a jackknife approach for ‘bootstrapping’, estimating a standard error and a Z score for the F_*ST*_ measurements. Regions of the genome with long-range LD listed in Table 1 of Price et al.,^86^ were excluded from F_*ST*_ analysis and we further utilized *smartpca’s* LD regression function to correct for remnants of LD prior to F_*ST*_ estimation.

### PCA, model-based clustering, and chromosome painting

Axes of genetic variation (principal components—PCs) were computed using *smartpca*. Ten PCs were computed with 10 outlier removal iterations (default = 5), while maintaining all other default parameters. PC plots and F_*ST*_ heatmaps showing the clustering of the populations into subgroups were generated using R 3.6.1^29^. To further investigate the population structure in our data set, we performed model-based clustering as implemented in Admixture^33^. Admixture utilizes a maximum likelihood approach to estimate the underlying ancestral coefficients and allele frequencies and then a moving block bootstrap approach for estimating standard errors. This analysis was performed on the set of “ancestry informative markers” described above, which were LD-pruned for MAF ≥ 0.05. The analysis was done with 5 cross-validation runs (K = 1–5) and 300 bootstrap runs. Also, the samples were sorted using Plink1.9′s *–indiv-sort* flag in the order BA-FO-SB so as to allow for proper grouping in the ancestral coefficients (Q) bar plot. We further resolved the clusters by running Structure v2.3.4^34^ in the admixture mode using 3 pre-defined clusters with 20,000 burnin and 100,000 main iterations. To explore the fine-scale genetic relatedness captured by haplotype structure, we ran ChromoPainter in the linked (LD) mode on a subset of 125 Cameroonian individuals (BA = 50, SB = 50, FO = 25) (as this haplotype-based method is powerful enough to capture similar information as it would in a large sample size, thereby, effectively reducing computational cost) obtained by thinning the full data-set using Plink1.9′s *–thin-indiv-count* command which does a random sampling of the number of samples required. ChromoPainter summarizes the genomic proportions shared among each donor and recipient individual as a “Coancestry matrix” (X_ij_) of the expected number of genetic elements donated to individual i from individual j^35^. We then used FineStructure to assign individuals into clusters setting 100,000 burnin and sampling iterations each. Finally, we generated a tree file with FineStructure for a better assignment of the individuals into different clusters. Clusters were visualized and cluster plots produced using the FineStructure graphical user interphase (fineStructureGUI) and R.

### Genome scan for signatures of selection

Signatures of selection were investigated by computing the integrated extended haplotype homozygosity (EHH) score (iHS) and cross-population locus-specific integrated (EHH) score (Rsb) using the R package REHHv3.01 (*rehh*)^51^. *rehh* estimates the probability that a locus, randomly shuffled from any chromosome is identical by descent to a core (focal) locus (ancestral/derived). As this probability breaks down with increasing distance upstream and downstream from the focal locus, the integral of the area under the EHH plot when the focal allele is an ancestral allele to the integral when the focal allele is a derived allele yields the iHS score. Therefore, large negative iHS values would signify selection at the derived allele and vice versa^87^. In the absence of a focal locus, Rsb is computed using EHH scores for all loci. Replacing the focal and derived alleles with the two populations being compared as popA and popB respectively, Rsb yields selection signatures for pairwise population comparisons. Thus, as above, large negative Rsb values would signify selection in popB and vice versa. iHS and Rsb statistics were computed in our data set using phased haplotypes with MAF ≥ 0.05. First, iHS was computed on the pooled data set (with all the ethnicities), then Rsb was computed with separate pairs of the different ethnicities (SBvsBA, SBvsFO, and BAvsFO). To assess the significance of selection signatures, *rehh* computes a two-sided *p*-value from the Gaussian cumulative distribution function of iHS estimates. The *p*-values were adjusted by the Benjamin–Hochberg (BH) and Bonferroni (BF) methods. Furthermore, *rehh* generates manhattan plots for visualization of iHS and Rsb results with their corresponding *p*-values. Genome-wide significance threshold for the manhattan plots was estimated by dividing 0.05 by the effective number of SNPs (those that passed all filters and were used to compute iHS) (Bonferroni method).

### Extended Lewontin–Krakauer Fst outlier test (FLK)

We further computed the extended Lewontin–Krakauer Fst outlier test (FLK)^46^ on a dataset of unlinked loci, LD-pruned to exclude pairs of SNPs with LD (r^2^) > 0.1 within 5 kb genomic regions with a step size of 50 bp using hapFLKv1.40^88^. FLK accounts for unequal effective population sizes (Ne) and hierarchical population structure by estimating a kinship matrix which is used to model the covariance matrix of the population allele frequencies. Since the FLK test is not robust to very low levels of heterozygosity, we excluded SNPs with ancestral allele frequency (AAF) < 0.2 as recommended^46^. FLK test for haplotypes (hapFLK)^88^ was performed per chromosome using linked loci with MAF ≥ 0.05. hapFLK screens for positive selection signatures using the Sheet and Stephens model^89^ to summarize local haplotype diversity in a sample by clustering similar haplotypes together. The clusters are then considered as alleles to compute the FLK statistic. A K-parameter (number of clusters to model) of 30 was used while all other options were set to default. Although the genome-wide distribution of hapFLK appears to be bimodal with a large proportion of the values showing good fit to a normal distribution, Fareillo et al., implemented a means of estimating *p*-values via robust estimators of mean and variance from a standard normal distribution, and reducing the influence of outliers, using a *rlm* function in R. The p-values were adjusted following the procedure previously described. Genome-wide significance threshold was computed as previously described. hapFLK manhattan plots were generated using the qqman package in R^45^.

### Ethical approval

All experiments in this study were conducted in adherence to the set of ethical principles of the Declaration of Helsinki. Ethical clearance for the study was obtained from the Institutional Review Board of the Faculty of Health Sciences, University of Buea (proposal number: ID D7.1.A/MPH/SWP/PDPH/PS.CH/2340/811) while administrative authorization was sought from the South West Regional Delegation of Public Health. Authorization to conduct the surveys in primary schools was obtained from the Regional Delegation of Basic Education or the Catholic Education Secretariat. Informed consent was obtained from each participant or their caregiver following a clear explanation of the content of the information sheet for the cases and blood bank donors used as controls. Authorization to enroll participants from health facilities or schools was obtained from the Director or Head teacher and only subjects/caregivers who volunteered to participate by signing a written informed consent were enrolled.

## Supplementary Information

Supplementary Information.

Supplementary Dataset 1.

Supplementary Dataset 2.

## Data Availability

Data used in this study are available at MalariaGEN’s Oxford Resource Center and data access and retrieval are granted according MalariaGEN’s policies via https://www.malariagen.net/. All scripts and software information including additional resources used in the analysis are present in the GitHub project (https://github.com/esohkevin/CamPopStruct).
