# Supplementary material for: Fine scale human genetic structure in three regions of Cameroon reveals episodic diversifying selection
Source: Sci Rep. 2021 Jan 13;11:1039. doi: 10.1038/s41598-020-79124-1 (PMC7807043; doi:10.1038/s41598-020-79124-1)
Supplement: Supplementary file 1 — Supplementary Information. [file 41598_2020_79124_MOESM1_ESM.docx]

# Fine scale human genetic structure in three regions of Cameroon reveals episodic diversifying selection

Kevin K. Esoh^1†^, Tobias O. Apinjoh^2†*^, Steven G. Nyanjom^1^, Ambroise Wonkam^3^, Emile R. Chimusa^3^, Lucas Amenga-Etego^4^, Alfred Amambua-Ngwa^5^, and Eric A. Achidi^2^

1. Department of Biochemistry, Jomo Kenyatta University of Agriculture and Technology, Nairobi, P.O. Box 62000 City Square, Kenya.
2. Department of Biochemistry and Molecular Biology, University of Buea, Buea, P.O. Box 63, South West Region, Cameroon.
3. Division of Human Genetics, Department of Pathology, Institute of Infectious Disease and Molecular Medicine, University of Cape Town, Health Sciences Campus, Anzio Rd, Observatory, 7925, South Africa.
4. West African Centre for Cell Biology of Infectious Pathogens, University of Ghana, Legon, Accra, Ghana
5. Medical Research Council Unit The Gambia at LSHTM, Banjul, The Gambia.

*Corresponding Author: [apinjoh.tobias@ubuea.cm](mailto:apinjoh.tobias@ubuea.cm)

† These authors contributed equally to this work

# Quality Control

Plink compares the sex assignment on the input data with the sex information extracted from computing the X chromosome inbreeding coefficients (F estimates). F estimates < 0.2 are called as females, while estimates > 0.8 are called as males. Since the pseudo-autosomal regions of the X chromosome can severely confound F estimates, they were removed prior to sex check using Plink1.9’s --split-x flag, while LD-pruning ensured that only 652,416 independent SNPs were used in this step. Twelve individuals, one from each pair with Pi HAT (IBD) > 0.1875 (halfway between expected IBD for third- and second-degree relatives) were removed, removing only the individual with a higher missingness (**Fig.S1**). After individual QC, a total of 1,185 individuals were retained for further analysis. A total of 1,714,262 SNPs passed all filters and were retained for further analyses.


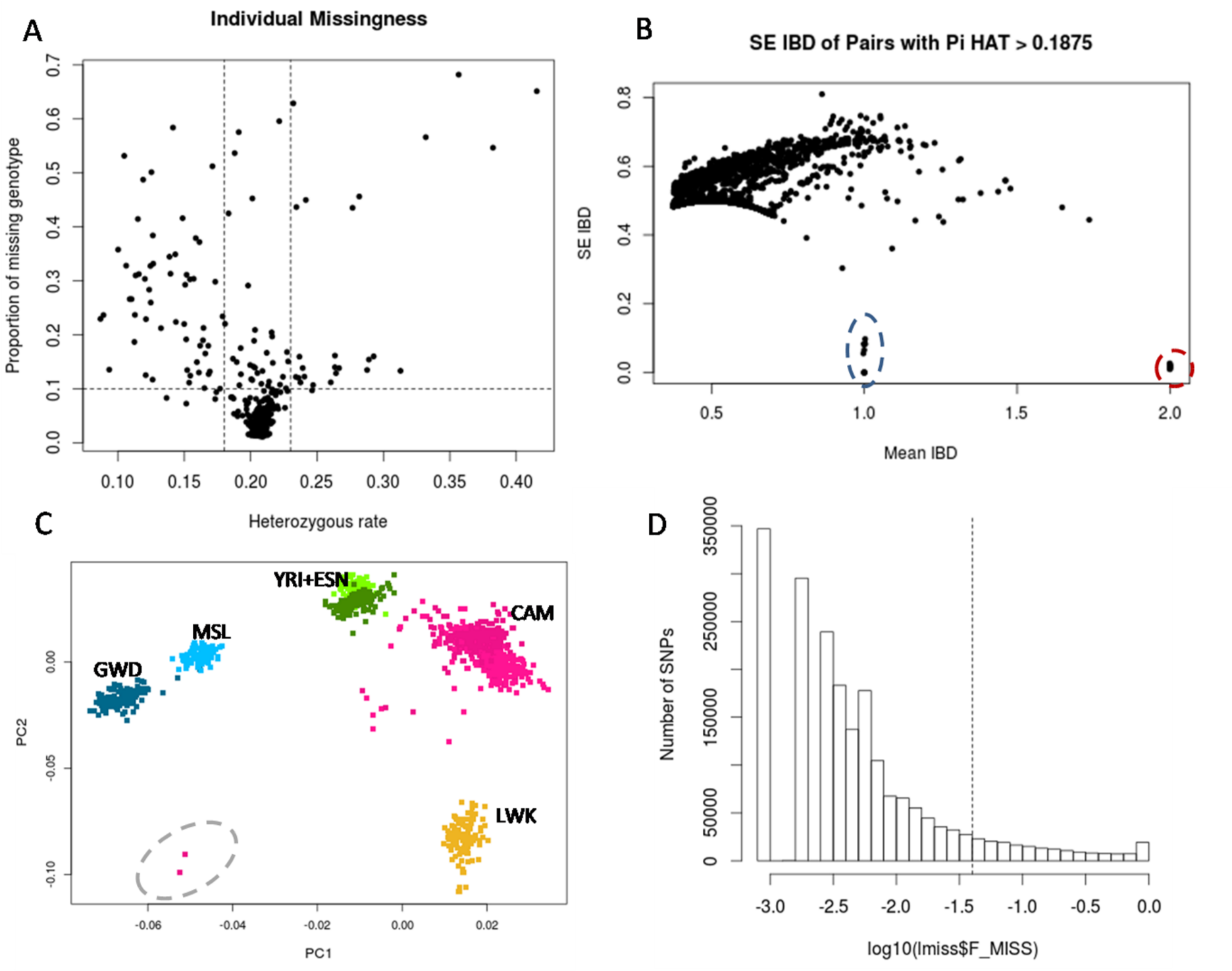


**Figure S1. Quality Control.** A) Individual missing data rate. Individuals with more than 10% missing values were removed. Also, individuals with heterozygosity < 0.180 and individuals with heterozygosity > 0.230 were excluded from further analysis. B) Identity-by-descent report for related individuals. Pi HAT is the mean IBD for pair of individuals. SE is the standard error (all computations provided in the R script where Z0 is the probability that a pair of individuals shares zero allele IBD; Z1 is the probability that they share 1 allele IBD and Z2 is the probability that they share 2 alleles IBD). The circled regions show samples with a high probability of sharing 1 or >1 alleles IBD. The red circles indicate duplicate samples (mean IBD > 1.98) (these were not homozygotic twins as there was no mean IBD value absolutely = 2). One of each individual with mean IBD > 0.1875 was excluded from further analysis. The choice for exclusion of an individual from a pair of duplicates was guided by their missing genotype rates. The individual with the higher missing rate was excluded. C) Projection of Cameroonian ethnicities against African populations from the 1000 Genomes reference panel showing ancestral outliers (gray broken circle). D) Proportion of SNPs by minor allele frequency. SNPs with MAF < 1% were removed. Studies have shown that GWASs for common variants association with disease are highly underpowered for rare variants. The plots were produced using *R* 3.6.1 ^1^

# PCA of Cameroonian populations


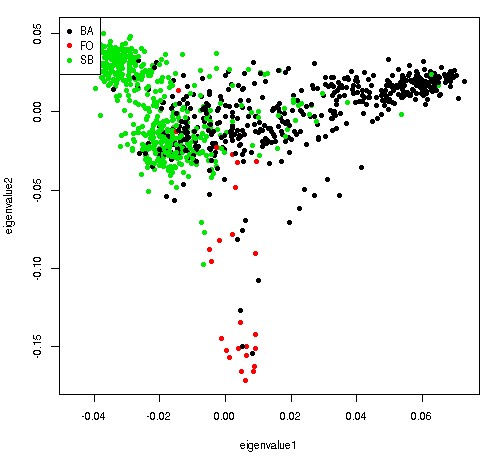


**Figure S2. PCA on Cameroonian populations.** Three clusters representing the three ethnicities are apparent based on PC1 and PC2. The plot was produced using *R* 3.6.1 ^1^.


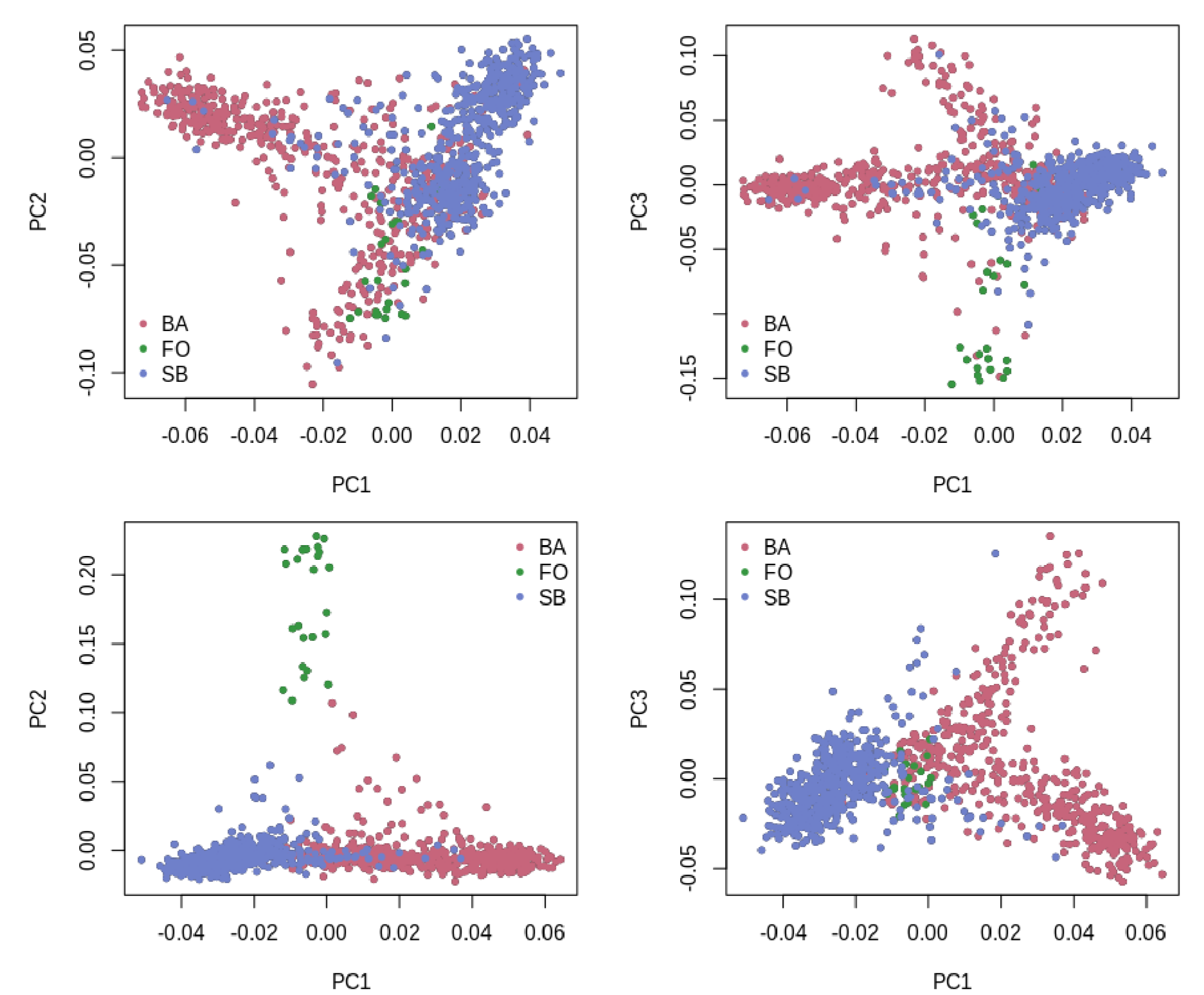

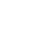


**A**


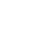


**D**


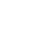


**C**


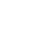


**B**

# Figure S3. PCA of Cameroonian ethnicities showing fine-structure. The plots were produced using R 3.6.1 ^1^.

# Generation of ancestry informative markers

Ancestry informative markers (AIMs) are loci that have substantial allele frequency differences across populations ^2.^ These markers can be used to assign individuals into clusters based on biological (ancestral) or geographical (continental) boundaries. These can thus be used to resolve structure in populations ^3^. Precalculated lists of AIMs exists. However, these mostly apply to crosscontinental populations ^4^. Furthermore, there are several measures of marker information content as listed in Table 1 of Rosenberg *et al* ^3^. We used the Fst measure in this study to generate AIMs by selecting all SNPs with Fst greater than or equal to the highest Fst (genetic distance) between the ethnicities studied. Fst between the BA and SB was 0.001 whereas Fst between the FO and both BA and SB was 0.003. Hence, AIMs were considered as SNPs with pairwise population Fst (Hudson) ≥ 0.003.

# Model-based clustering by Structure (v2.3.4)


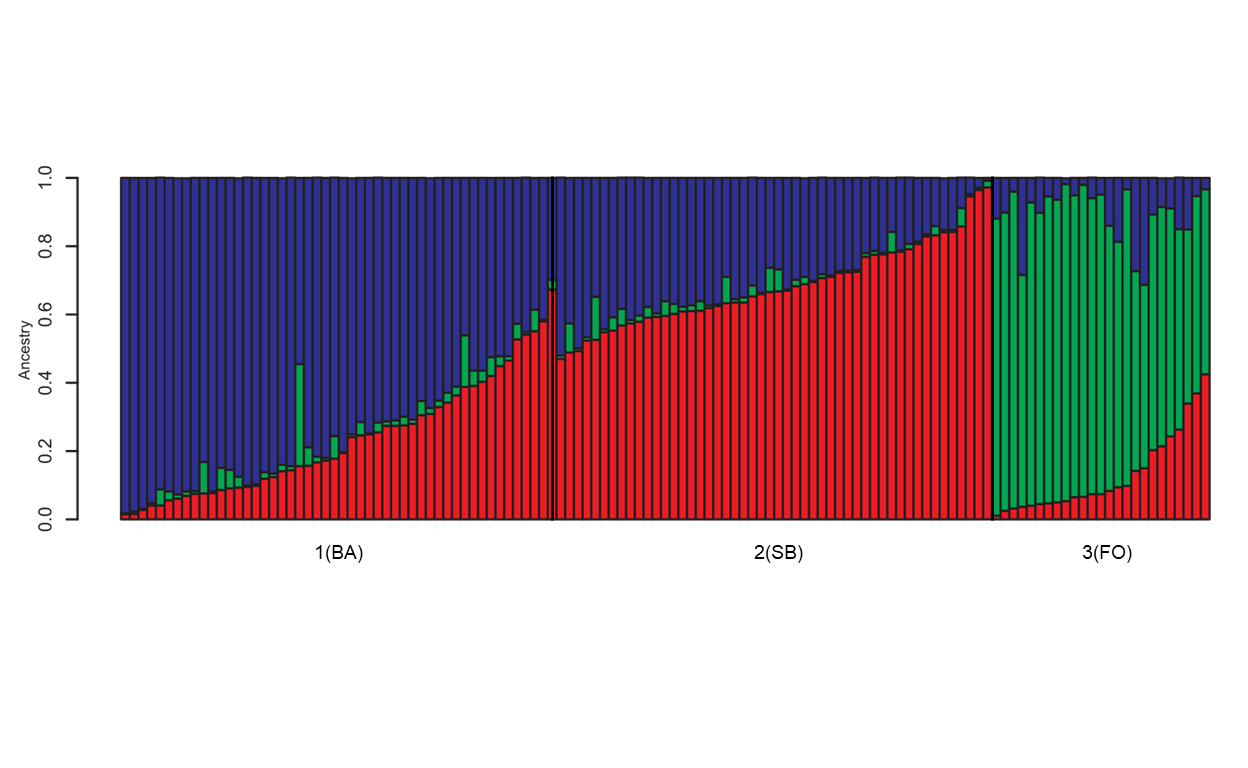


# Figure S4. Structure output of ancestral proportions among Cameroonian ethnicities

An admixture model with 3 pre-defined clusters was run with 50 BA, 50 SB, and 25 FO individuals setting 20,000 burnin iterations and 100,000 main iterations. The clusters were sorted according to population ID (1=BA, 2=SB, 3=FO). The BA population harbored ~73.3% basal Bantu ancestry, the SB harbored ~68.4% of a basal Semi-Bantu ancestry, while the FO harbored ~76.4% likely non-Cameroonian ancestry. The plot was produced using R 3.6.1 ^1^.

**Genome scan by the extended Lewontin-Krakauer Fst outlier statistic (FLK)**


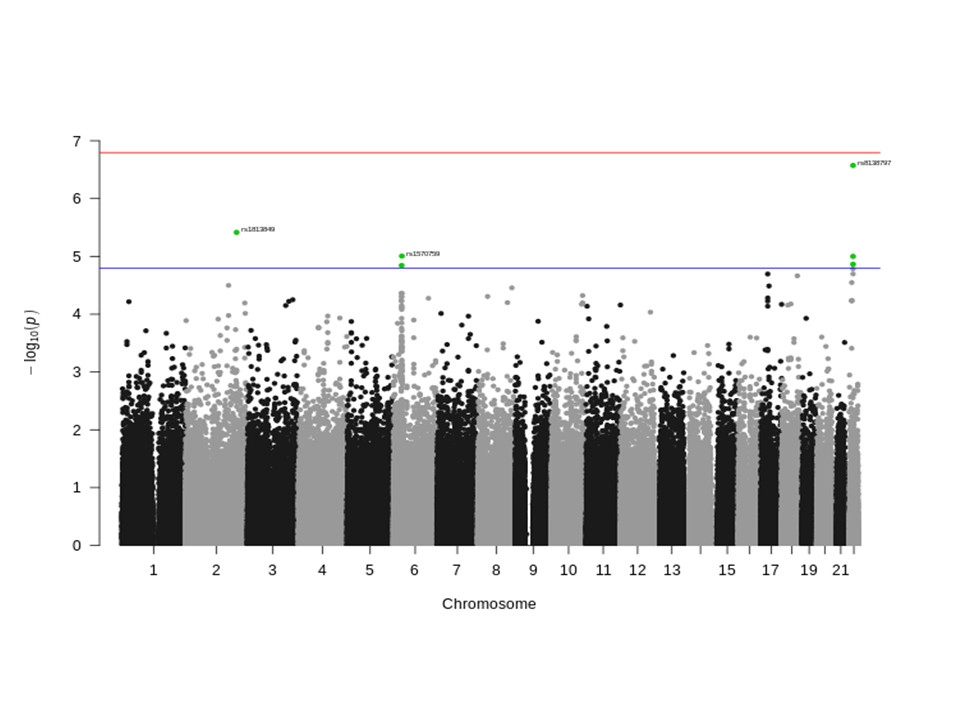


# Figure S5. Manhattan plot of FLK p-values The plot was generated using the R package *qqman* ^5^.

# Table S1: FLK statistic sorted by flk column from largest (top) to smallest (bottom)

| **rsid** | **chr:pos** | **ref** | **alt** | **SB** | **BA** | **FO** | **flk** | **p-value** | **BH_adj_**  **P** | **Bonf** |
| --- | --- | --- | --- | --- | --- | --- | --- | --- | --- | --- |
| **rs8138797** | **22:31044286** | G | A | 0.49 | 0.62 | 0.76 | **30.28** | **2.68e-07** | **0.083** | **0.083** |
| rs1813849 | 2:200142847 | T | C | 0.78 | 0.79 | 0.44 | 24.94 | 3.85e-06 | 0.516 | 1 |
| rs1570759 | 6:33633940 | G | A | 0.83 | 0.73 | 0.84 | 23.05 | 9.87e-06 | 0.516 | 1 |
| rs734479 | 22:31043484 | A | G | 0.41 | 0.53 | 0.64 | 23.02 | 1.00e-05 | 0.516 | 1 |
| rs2267162 | 22:31010997 | C | T | 0.46 | 0.56 | 0.74 | 22.40 | 1.37e-05 | 0.516 | 1 |
| rs9275273 | 6:32662559 | C | A | 0.58 | 0.70 | 0.5 | 22.31 | 1.44e-05 | 0.516 | 1 |
| rs16988927 | 22:31042490 | G | A | 0.41 | 0.53 | 0.62 | 22.03 | 1.64e-05 | 0.516 | 1 |
| rs2283873 | 22:31013296 | G | A | 0.46 | 0.56 | 0.74 | 21.63 | 2.01e-05 | 0.516 | 1 |
| rs11080058 | 17:26742060 | G | A | 0.73 | 0.64 | 0.46 | 21.62 | 2.02e-05 | 0.516 | 1 |
| rs386804130 | 18:61375975 | G | A | 0.65 | 0.54 | 0.76 | 21.48 | 2.17e-05 | 0.516 | 1 |
| rs637629 | 22:25908441 | A | G | 0.55 | 0.67 | 0.56 | 20.94 | 2.83e-05 | 0.516 | 1 |
| rs6724543 | 2:168756871 | A | G | 0.73 | 0.80 | 0.52 | 20.72 | 3.17e-05 | 0.516 | 1 |
| rs12453488 | 17:31897097 | G | A | 0.61 | 0.48 | 0.44 | 20.67 | 3.25e-05 | 0.516 | 1 |
| rs16904656 | 8:133330138 | G | C | 0.70 | 0.71 | 0.36 | 20.52 | 3.50e-05 | 0.516 | 1 |
| rs9273012 | 6:32611641 | A | G | 0.67 | 0.76 | 0.54 | 20.08 | 4.35e-05 | 0.516 | 1 |
| rs9272783 | 6:32610378 | C | T | 0.67 | 0.76 | 0.54 | 20.08 | 4.35e-05 | 0.516 | 1 |
| rs9272219 | 6:32602269 | G | T | 0.67 | 0.76 | 0.54 | 20.08 | 4.35e-05 | 0.516 | 1 |
| rs4752448 | 10:122405330 | A | C | 0.79 | 0.81 | 0.5 | 19.91 | 4.75e-05 | 0.516 | 1 |
| rs6985355 | 8:38588313 | G | A | 0.66 | 0.73 | 0.42 | 19.83 | 4.93e-05 | 0.516 | 1 |
| rs6457622 | 6:32664163 | A | C | 0.60 | 0.71 | 0.52 | 19.83 | 4.98e-05 | 0.516 | 1 |

# Derive allele distribution of selected alleles in Cameroonian ethnicities


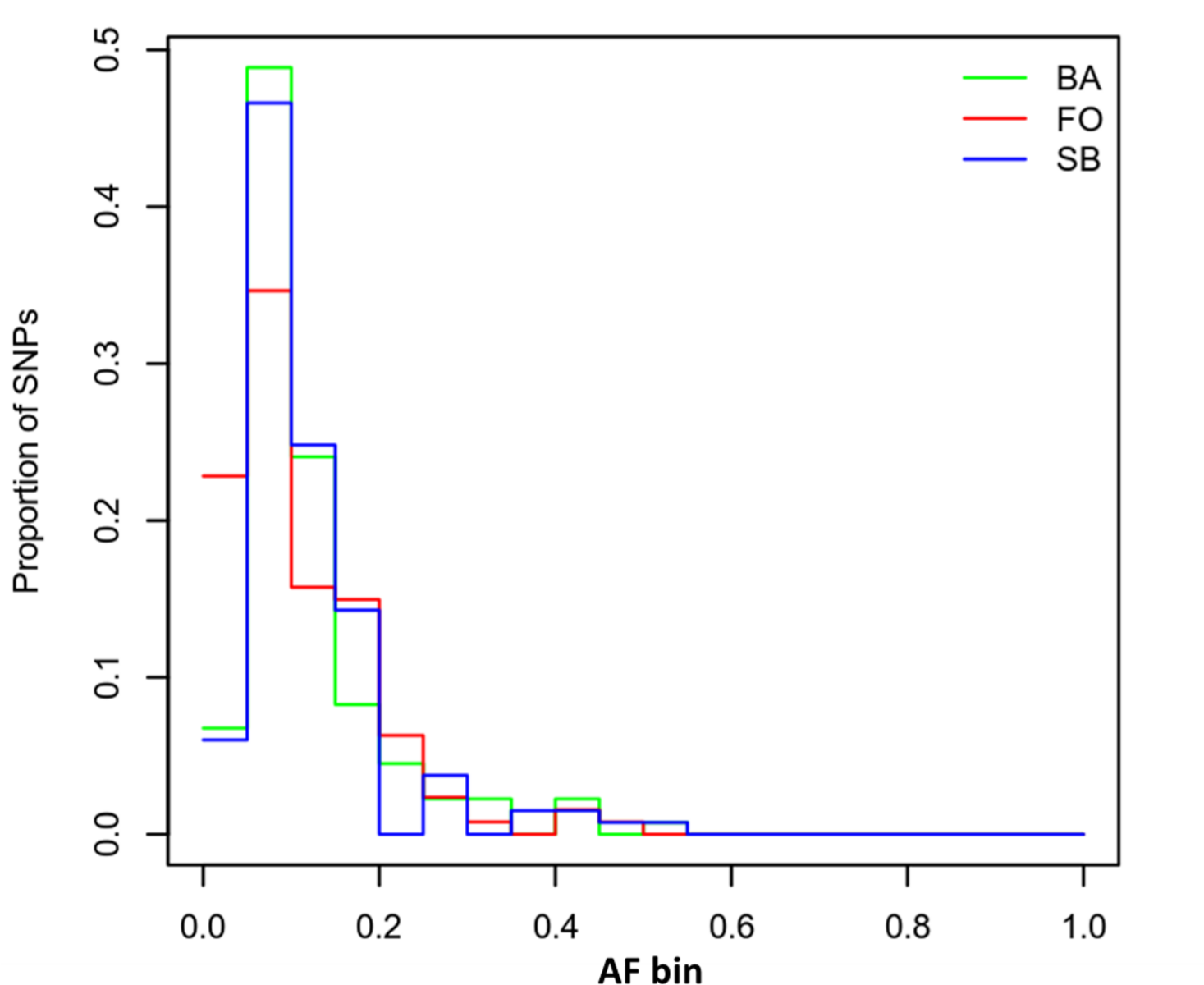


# Figure S6. iHS Derived AF Spectrum

A cluster-stratified analysis of the allele frequency distribution of the derived alleles at selected loci showed great heterogeneity in the selection pattern among the ethnic groups. This further supports data that showed differently selected loci per population (Rsb results). R 3.6.1 was used for plotting ^1^.

# Allele frequency distribution demonstrates genetic differentiation among Cameroonian and continental populations

One useful indicator of population differentiation is the spectrum of allele frequencies (AFs) which are usually particularly sensitive to changes in populations as rapid expansions or contractions, strong selective sweeps, and bottlenecks. Based on a cluster-stratified AF analysis (*bin size* = 0.05) among randomly selected Cameroonian individuals (BA=50, SB=50, FO=25) using Plink1.9’s *-within* command, we observed a substantial difference in low frequency alleles of the FO from the BA and SB populations, who in turn had similar AF spectra (**Fig.S7a**). In addition, the BA and SB had a higher proportion of rare alleles than the FO. After SNP ascertainment in the Mende population from Sierra Leone (see Methods) population and inclusion of selected populations from the 1KGP3 (MSL, LWK, CEU and CHB), we observed considerable differences in AF spectra among continental populations, and particularly at low AF bins (**Fig.S7b**). All African populations demonstrated similar spectra as expected, with an excess of low frequency derived alleles consistent with recent population expansion and/or strong positive selective sweeps or weak purifying selections affecting multiple genomic loci ^6^. However, the FO population of Cameroon deviated from other African populations at low frequency bins (0.05 – 0.1). While this may be indicative of the presence of substantial amounts of non-African ancestral proportions in the population, it may well be due to decreased gene flow/admixture with other Cameroonian/African populations, as well as sustained inbreeding within the population reducing genetic diversity. At the continental scale, the Han Chinese population from Beijing (CHB) demonstrated the lowest proportion of low frequency derived alleles as has been previously reported ^6^ followed by the CEU population with European ancestry, both cases consistent with the widely accepted “out-of-Africa” hypothesis. These populations are believed to have been founded by a small group of anatomically modern humans that experienced a bottleneck migrating out of Africa ^7,8^, such that their allele pool, and consequently genetic diversity were dramatically reduced.


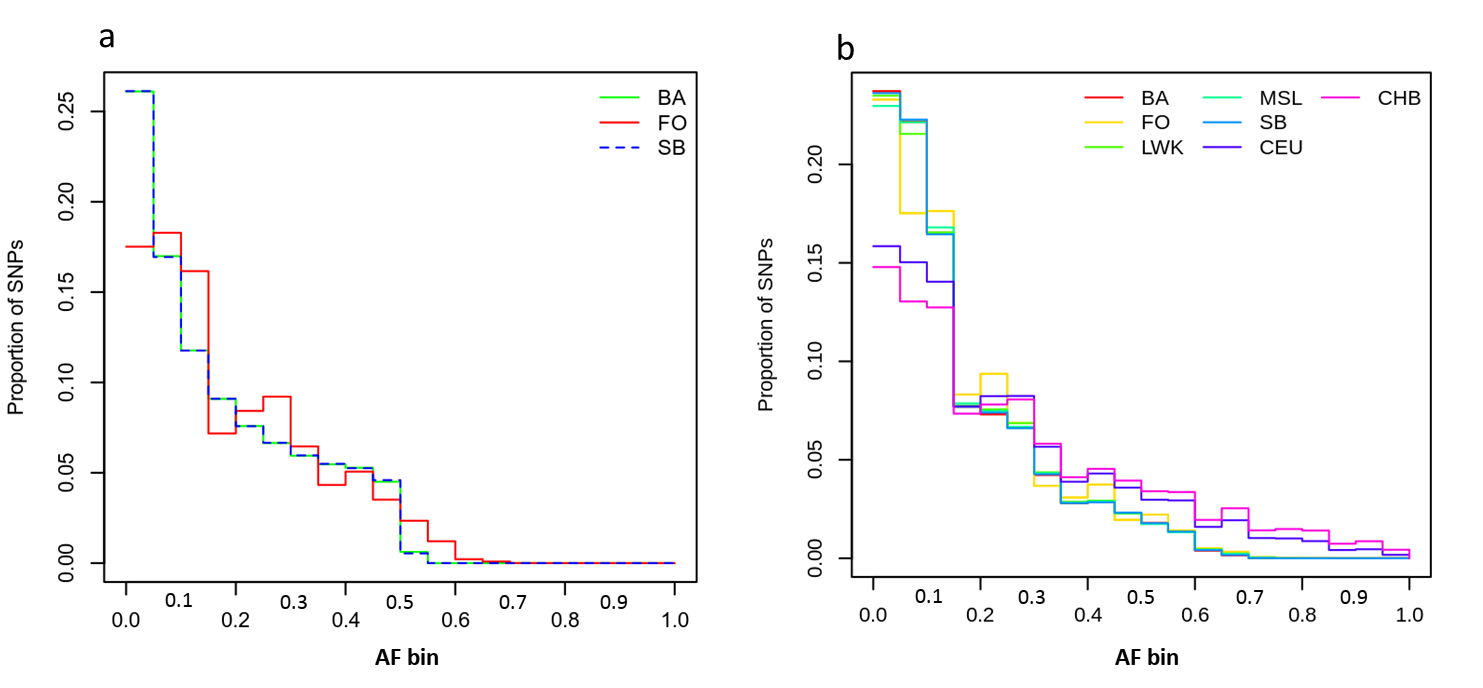


**Figure S7**. **Allele Frequency Spectrum**. a) Allele frequency spectrum among Cameroonian ethnic groups. The blue line (SB) and green line (BA) are perfectly overlayed such that the blue line is broken to reveal the green. b) Derived allele frequency spectrum of Cameroonian and selected populations from the 1000 Genomes phase 3 reference panel We used R 3.6.1 for plotting ^1^.

**Cross**

**-**

**Population genome scan and**

**haplotype variant of FLK (hapFLK)**


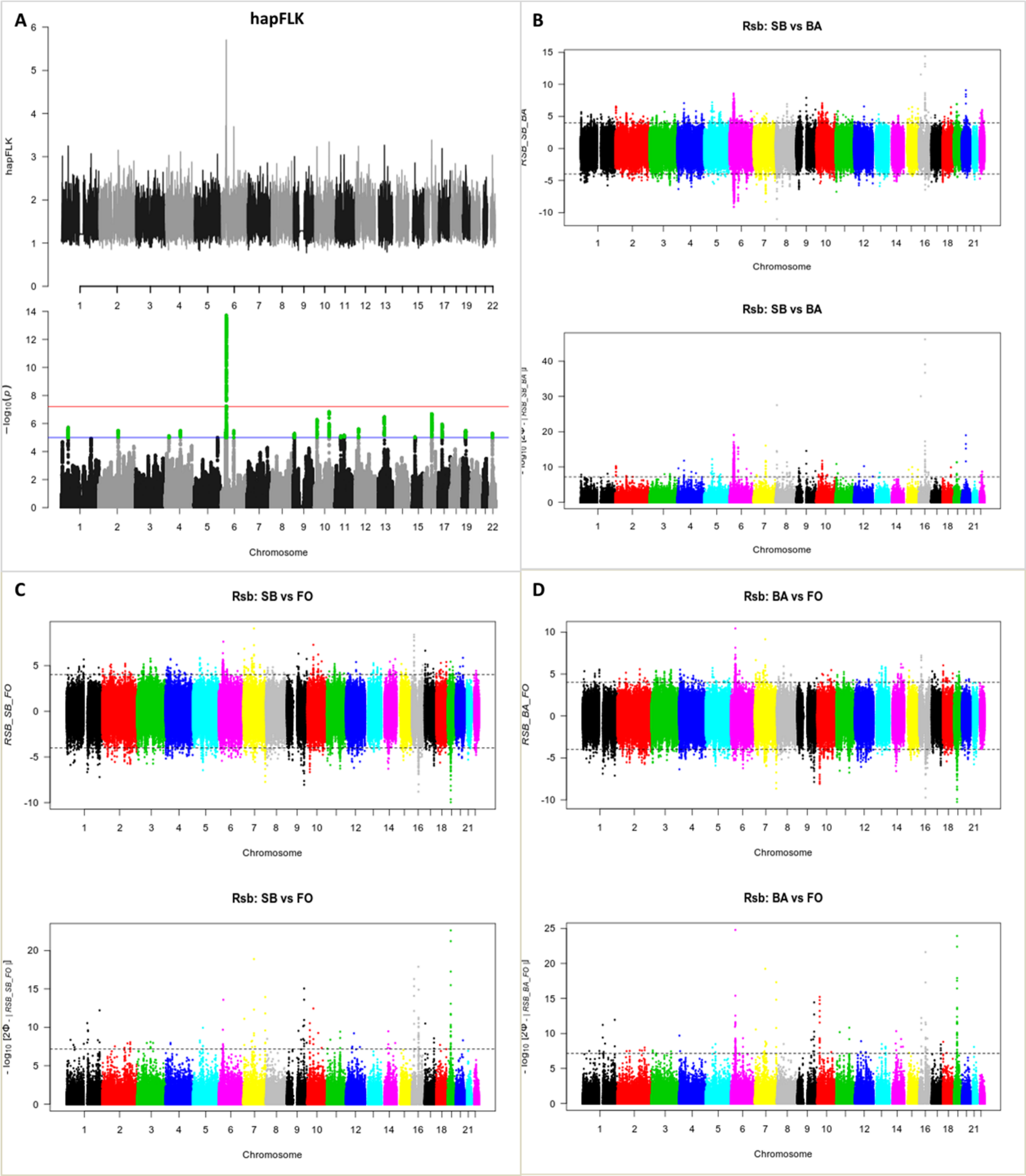


# Figure S8. HapFLK and corresponding –log10 *p*-values (A) and Cross-Population selection scan by Rsb (B - D). All plots were generated using *qqman* ^5^.

**References**

1. R Core Team. R: A Language and Environment for Statistical Computing. *R Foundation for. Statistical. Computing. Vienna, Austria* (2019). url:https://www.r-project.org

2. Shriver, M. D. *et al.* Skin pigmentation, biogeographical ancestry and admixture mapping. *Hum. Genet.* **112**, 387–399 (2003).

3. Rosenberg, N. A., Li, L. M., Ward, R. & Pritchard, J. K. Informativeness of Genetic Markers for Inference of Ancestry. *Am. J. Hum. Genet.* **73**, 1402–1422 (2003).

4. Huckins, L. M. *et al.* Using ancestry-informative markers to identify fine structure across 15 populations of European origin. *Eur. J. Hum. Genet.* **22**, 1190–1200 (2014).

5. D. Turner, S. qqman: an R package for visualizing GWAS results using Q-Q and manhattan plots. *J. Open Source Softw.* **3**, 731 (2018).

6. Keinan, A., Mullikin, J. C., Patterson, N. & Reich, D. Measurement of the human allele frequency spectrum demonstrates greater genetic drift in East Asians than in Europeans. *Nat. Genet.* (2007). doi:10.1038/ng2116

7. Keinan, A. & Clark, A. G. Recent explosive human population growth has resulted in an excess of rare genetic variants. *Science.* **336**, 740–743 (2012).

8. López, S., van Dorp, L. & Hellenthal, G. Human dispersal out of Africa: A lasting debate. *Evolutionary Bioinformatics* **11**, 57–68 (2015).

# Figure Legends

**Figure 1. Pairwise Fst and PCA analysis of Cameroonian and world populations**

(a) Clustered heatmap showing genetic distance by pairwise population F*_ST_* (Hudson) estimation. AFR=African, EAS=East Asian, EUR=European, AMR=American and SAS=South Asian ancestry. The red color denotes closely related population, hence low F*_ST_* while the decrease in redness to yellow represents increasing genetic distance (high F*_ST_*). Five clusters are apparent corresponding to the five continental populations (distinguished broadly by five colors) in the 1000 Genomes project. (b) PCA of Cameroonian populations with the 1000 Genomes populations. Cameroonian populations clustered within African populations

**Figure 2.** **PCA of Cameroonian populations and other Africa populations**

(a) Cameroonian populations with other African populations produce a clustering pattern correlated with geography where West African populations (GWD, MSL, ESN, YRI) clustered to the West, the lone east African population (LWK) clustered to the East, while Cameroonian populations clustered West-Centrally. (b) PCA for Cameroonian ethnicities only. PC1 and PC2 separate the three ethnicities, (c) PC1 and PC3 separate the Bantu and Semi-Bantu.

**Figure 3. Model-based clustering and Coancestry estimation.** (a) Model-based clustering cross-validation (CV) error. Lowest CV error recorded at k=3 indicating three clusters. (b) and (c) show ancestral proportions Q, for each ethnicity colored using the RBG color scheme. (d) Coancestry estimation by FineStructure.

**Figure 4.** **iHS and corresponding –log10(p-values) Manhattan plots**.

(a) iHS plot for the autosomes. Negative values signify selection on derived alleles while positive values are associated with selection on ancestral alleles. (b) Distribution of iHS values as observed in the populations (blue) and as expected under neutral evolution (red). Lower plot represents quantile-quantile (Q-Q) plot of iHS p-values. The plot shows that the test statistics are not inflated.

**Figure 5. Manahattan Plot of hapFLK p-values**. Genome-wide significance threshold (red line), suggestive threshold (blue line).

**Figure S1. Quality Control.** A) Individual missing data rate. Individuals with more than 10% missing values were removed. Also, individuals with heterozygosity < 0.180 and individuals with heterozygosity > 0.230 were excluded from further analysis. B) Identity-by-descent report for related individuals. Pi HAT is the mean IBD for pair of individuals. SE is the standard error (all computations provided in the R script where Z0 is the probability that a pair of individuals shares zero allele IBD; Z1 is the probability that they share 1 allele IBD and Z2 is the probability that they share 2 alleles IBD). The circled regions show samples with a high probability of sharing 1 or >1 alleles IBD. The red circles indicate duplicate samples (mean IBD > 1.98) (these were not homozygotic twins as there was no mean IBD value absolutely = 2). One of each individual with mean IBD > 0.1875 was excluded from further analysis. The choice for exclusion of an individual from a pair of duplicates was guided by their missing genotype rates. The individual with the higher missing rate was excluded. C) Projection of Cameroonian ethnicities against African populations from the 1000 Genomes reference panel showing ancestral outliers (gray broken circle). D) Proportion of SNPs by minor allele frequency. SNPs with MAF < 1% were removed. Studies have shown that GWASs for common variants association with disease are highly underpowered for rare variants. The plots were produced using *R* 3.6.1 ^1^

**Figure S2. PCA on Cameroonian populations.** Three clusters representing the three ethnicities are apparent based on PC1 and PC2. The plot was produced using *R* 3.6.1 ^1^

**Figure S3. PCA of Cameroonian ethnicities showing fine-structure**. The plots were produced using R 3.6.1 ^1^

**Figure S4. Structure output of ancestral proportions among Cameroonian ethnicities**

**Figure S5. Manhattan plot of FLK p-values**. The plot was generated using the R package *qqman* ^5^

**Figure S6. iHS Derived AF Spectrum**

**Figure S7**. **Allele Frequency Spectrum**. a) Allele frequency spectrum among Cameroonian ethnic groups. The blue line (SB) and green line (BA) are perfectly overlayed such that the blue line is broken to reveal the green. b) Derived allele frequency spectrum of Cameroonian and selected populations from the 1000 Genomes phase 3 reference panel We used R 3.6.1 for plotting ^1^.

**Figure S8. HapFLK and corresponding –log10 *p*-values**. (A) and Cross-Population selection scan by Rsb (B - D). All plots were generated using *qqman* ^5^

**Table Legend**

**Table 1. Variants with strong signatures of selection in coding genomic regions sorted by ihs scores from most negative (top) through least negative to most positive (bottom).** rsid, Reference SNP ID; chr:pos, Chromosome number and position; ref, Reference allele; alt, Alternate allele; alt.AF, Alternate allele frequency; ihs, Integrated haplotype score; p-value (bh), Benjamin–Hochberg adjusted p-value; a.a change, Amino acid change.

**Table S1: FLK statistic sorted by flk column from largest (top) to smallest (bottom).** rsid, Reference SNP ID; chr:pos, Chromosome number and position; ref, Reference allele; alt, Alternate allele; alt.AF, Alternate allele frequency; ihs, Integrated haplotype score; p-value (bh), Benjamin–Hochberg adjusted p-value; a.a change, Amino acid change.

**Data S1:** Pairwise F*_ST_* among Cameroonian populations and populations from the 1000 Genomes Phase 3 reference panel.

**Data S2**: List of variants (133) with significant iHS values sorted by iHS scores from most negative (top) through least negative to most positive (bottom), and Variant Effect Predictor- and BioMart-annotated gene list.
